# Suppression of yolk formation, oviposition and egg quality of locust (*Locusta migratoria manilensis*) infected by *Paranosema locustae*

**DOI:** 10.3389/fimmu.2022.848267

**Published:** 2022-07-21

**Authors:** Yao-Wen Hu, Shao-Hua Wang, Ya Tang, Guo-Qiang Xie, Yan-Juan Ding, Qing-Ye Xu, Bin Tang, Long Zhang, Shi-Gui Wang

**Affiliations:** ^1^ Hangzhou Key Laboratory of Animal Adaptation and Evolution, College of Life and Environmental Sciences, Hangzhou Normal University, Hangzhou, China; ^2^ Department of Entomology, China Agricultural University, Beijing, China

**Keywords:** immunity, reproduction, Locusta migratoria manilensis, Paranosema locustae, trade-off

## Abstract

*Locusta migratoria manilensis* is one of the most important agricultural pests in China. The locust has high fecundity and consumes large quantities of food, causing severe damage to diverse crops such as corn, sorghum, and rice. Immunity against pathogens and reproductive success are two important components of individual fitness, and many insects have a trade-off between reproduction and immunity when resources are limited, which may be an important target for pest control. In this study, adult females *L. migratoria manilensis* were treated with different concentrations (5 × 10^6^ spores/mL or 2 × 10^7^ spores/mL) of the entomopathogenic fungus *Paranosema locustae*. Effects of input to immunity on reproduction were studied by measuring feeding amount, enzyme activity, vitellogenin (Vg) and vitellogenin receptor (VgR) production, ovary development, and oviposition amount. When infected by *P. locustae*, feeding rate and phenol oxidase and lysozyme activities increased, mRNA expression of *Vg* and *VgR* genes decreased, and yolk deposition was blocked. Weight of ovaries decreased, with significant decreases in egg, length and weight.Thus, locusts used nutritive input required for reproduction to resist invasion by microsporidia. This leads to a decrease in expression of *Vg* and *VgR* genes inhibited ovarian development, and greatly decreased total fecundity. *P. locustae* at 2 × 10^7^ spores/mL had a more obvious inhibitory effect on the ovarian development in migratory locusts. This study provides a detailed trade-off between reproduction and immune input of the female, which provides a reliable basis to find pest targets for biological control from those trade-off processes.

## Introduction

*Paranosema* (*Nosema*) *locustae* is an entomopathogenic fungus that only infects locusts. Since the 1980s, the fungus has shown great potential as a biological control in locust control (1, 2). Locusts infected by *P. locustae* can spread the pathogen to other nearby locusts through horizontal transmission. The fungus can also be transmited vertically, with microsporidia infecting offspring through locust eggs (3, 4). Recently, a study has found that microsporidia can also be transmitted through the feces of predators (5). After consumption by locusts, microsporidian spores germinate in the midgut lumen by everting the polar filament into cells, and the infection occurs through the sporoplasm to primarily infect the fat body (6). Microsporidia proliferate in the fat body and destroy normal host cell metabolism by capturing ATP and secreting hexokinase and hydrolase to deplete host glycogen (7–9). To resist the processes associated with infection, locusts must invest large amounts of energy.

Vitellogenesis, an imperative event of insect reproduction, is essential for insect fecundity (10, 11).In insects, vitellogenesis is a complex process involving two parts. First, vitellogenins (Vg) are biosynthesized in the fat body and released into the haemolymph. Maturing oocytes then absorb the vitellogenins from the haemolymph through endocytosis mediated by vitellogenin receptors (VgR) and accumulate yolk to form a mature egg, which increases in size as it matures (11, 12). In insects, juvenile hormone, insulin (or insulin-like peptide), and amino acid/target of rapamycin signaling pathways control vitellogenesis (13–16). Insulin and target of rapamycin signalling pathways regulate the terminal stages of oocyte development by regulating expression of vitellogenin (14). Total number of eggs produced and number of eggs in a single egg pod are affected by body size and life history of each individual (17). In *L. migratoria manilensis*, egg yolk biosynthesis is controlled by juvenile hormone (18) and is also dependent on feeding temperature. Vitellogenin first appears in the serum of adult females 5–9 days after emergence, with vitellogenin titer reaching 25 to 30 mg/mL in the second cycle of vitellogenin production (19).

In the face of predation, disease, and other hazards, the energy intake of an organism is generally insufficient for growth, reproduction, survival, and other important activities, which leads to trade-offs in allocation of resources (20, 21). Immunity and reproduction, are two critical processes that require a substantial investment of resources in female insects. Given that life-history evolution tends to optimize rather than maximize energy resources, reproduction and immunity can be mutually constraining (22). In *Alloallomobilus socius*, the mating process leads to a decrease in hemocyte load, lytic activity, and encapsulation and thus an increase in mortality (23). *P. locustae* infection in *L. migratoria manilensis* results in increased expression of defense genes, such as those for antimicrobial peptides, peroxiredoxin, and amine oxidase, increased investment in immunity, and consumption of energy materials for reproduction (2). In addition, microsporidia can inhibit phenol oxidase (PO) activity in locusts, which makes host melanization of pathogens difficult (2). In Orthoptera, crickets are frequently used as models to study the trade-off between immunity and reproduction.

The orthopteran *L. migratoria manilensis*, can be divided into aggregation and dispersion types, depending on their population density (24, 25). Locusts are a model orthopteran (26). However, they are also notorious because of the threat of mass migrations and are one of the most destructive agricultural pests worldwide (27). High locust fecundity is the basis of outbreaks. Plagues of locusts have been a historical problem in China and elsewhere. After nearly half a century of overuse of chemical pesticides, detrimental effects on environment have led to the recognition that more environmentally sound methods of pest control are needed (28). The use of *P. locustae* is one potentially sustainable approach to control locusts. The first report of microsporidia as a biological control tool was from the United States Department of Agriculture, which used *Nosema acidophagus* (29). *P. locustae* was isolated from locusts by Canning (30), and Henry (31) used *Melanoplus bivittatus* as a new host to increase spore numbers. Since then, *P. locustae* has since been used in locust control with remarkable success as a commercial, environmentally safe pest management tool. However, the continuous use of *P. locustae* has led to locusts developing a certain level of immunity. With the establishment of transgenic crops, the search for new gene targets is also an important research direction. In this study, the potential trade-off in investment between reproduction and immunity in locusts was explored. This study provides an important foundation to increase understanding of mechanisms of the trade-offs between insect immunity and other physiological functions and also to identify new potential targets for biological control.

## Materials and methods

### *L. migratoria manilensis* and *P. locustae*

Colonies of *L. migratoria manilensis* were maintained in the Key Laboratory of Animal Adaptation and Evolution at the Life and Environmental Science School of Hangzhou Normal University (Hangzhou, China). The feeding temperature was set at 30 ± 2°C with a photoperiod L16: D8 and 50% relative humidity. Locusts were hatched in small, clean boxes (10 cm × 15 cm × 20 cm) and then released into large, dry, well-ventilated cages of 50 cm × 50 cm × 50 cm at a density of 200 to 300 insects per cage. Locusts were provided fresh wheat seedlings and wheat bran for at least two generations before experimentation. A suspension of *P. locustae* provided by the China Agricultural University (Beijing, China) was diluted to either 5 × 10^6^ spores/mL or 2 × 10^7^ spores/mL and maintained at 4°C.

### Treatment design and infection of *L. migratoria manilensis*

Fresh wheat seedlings were divided into three equal portions of 100g each. One portion was soaked with 5 × 10^6^ spores/mL and another with 2 × 10^7^ spores/mL of *P. locustae*, and both were dried at 37°C for 10 min. The third portion was used as a control treatment. Food was provided from emergence, and on days 1–4 post emergence, the average daily weight of wheat seedlings consumed was recorded. The three groups of locusts were reared in the same conditions, and males and females were paired. Each treatment included three biological replications of five locust individuals each.

### PO and lysozyme activity in *L. migratoria manilensis*

There were three biological replicates of *P. locustae*-infection and control groups, with five individuals per replicate. In the three groups of locusts, fat bodies and ovaries were dissected at 2, 4, 6, 8, 10, 12, 14, 16, 18, and 20 d. Fat bodies and ovaries were frozen with liquid nitrogen and stored at −80°C.

To detect PO activity, 2 µL of serum was diluted to 20 µL using 0.9% normal saline, followed by addition of 40 μL of CAC buffer and 60 μL of 3 g/L L-dopa. Solutions were mixed and placed in a 25°C water bath for 30 min, and then, the OD_595_ nm value was determined. In a blank control, 60 μL of CAC buffer and 60 μL of 3 g/L L-dopa were used. Detection time of PO activity was 30 min. Total protein content was determined using a Coomassie bright blue protein kit following the manufacturer’s instructions (Nanjing Jiancheng Biological Company, Nanjing, China). The OD_495_ value was calculated as sample OD_495_ value − control OD_495_ value. One unit of PO activity (U) was defined as U = OD_495_ value/min/mg protein.

The detection method for lysozyme activity was adjusted according to Hultmark (32). To prepare of the bacterial suspension, micrococcus dry powder (Nanjing Jiancheng Biological Company, Nanjing, China) was used as the substrate, 0.1 mol/L phosphoric acid buffer (pH 6.4) was used as the substrate solvent and activity was confirmed when the 570 nm light absorption value was greater than 0.35. To detect lysozyme activity, 200 μL of bacterial suspension was removed and placed in an ice bath for 10 min. Then, 4 μL of locust serum added and mixed, and a UV2000 was used to determine the absorbency (A) at 570 nm. A sample was placed in a 37°C water bath for 30 min, moved to ice to stop the reaction for 10 min, and the absorption value at 570 nm was determined (A_0_). Lysozyme activity (U) was calculated as U = (A_0_ − A)/A.

### RNA extraction, cDNA synthesis and quantification of mRNA expression

From fat bodies and ovaries dissected imultaneously with collection of serum, total RNA was extracted using the TRIzol reagent (Invitrogen, Carlsbad, CA, USA) following the manufacturer’s instructions. Each treatment included three biological replicates of five locust individuals each. Dissecting time was 2, 4, 6, 8, 10, 12, 14, 16, 18, and 20 d after emergence. The RNA concentration was determined by measuring the absorbance at 260 nm with a spectrophotometer. First-strand cDNA synthesis was performed using a PrimeScript^®^ RT Reagent Kit with gDNA Eraser (Takara, Dalian, China) following the manufacturer’s protocol. All primers were designed to determine expression of the genes *VgA*, *VgB*, *VgR1*, and *VgR2* (**Table 1**). Expression levels of genes were normalized using the expression of actin. Reverse-transcription quantitative real-time PCR (RT-qPCR) was performed using a Bio-Rad CFX96 Real-Time PCR Detection System (Bio-Rad, Hercules, CA, USA) and Premix Ex Taq (SYBR Green) reagents (Takara). The 20 μL RT-qPCR reactions were subject to a thermal profile of 95°C for 3 min and then 40 cycles of 95°C for 10 s and 60°C for 30 s. The thermal melting profile was assessed using a final PCR cycle of 95°C for 30 s, with temperature increasing continuously from 60°C to 95°C. Relative gene expression levels were calculated using the 2^–ΔΔCT^ method, with three replicates per sample.

**Table 1:** RT-qPCR Primers.

Primer name	Primer sequence (5’–3’)	Amplified fragment length	Annealing temperature
VgA-F	CTTCAACTTTGCCCTCCGTG	194 bp	59°C
VgA-R	GCTTCAACTTATCTGCCAATCGT		
VgB-F	ATTAACGCCCTTTCACAGTCG	153 bp	59°C
VgB-R	TTCCTGGAGGTATTCTTTGTTGG		
VgR1-F	GGGAGGACTTGATTTACTGGACT	114 bp	59°C
VgR1-R	GGGCTATTTCTGTTGGCTTTC		
VgR2-F	TGACTCCAAAGTGAAGAAGACC	196 bp	59°C
VgR2-R	ATTGCCCTGTAGCACCATT		
LM-Actin-F	CCTGCCTCATGCCATTCTCA	110 bp	59°C
LM-Actin-R	CTCGCTCGGCTGTGGTAGTG	110 bp	59°C

### Paraffin sectioning of ovaries

In controls and groups infected with *P. locustae*, ovarian tissue sections were prepared by a paraffin method. Female locusts 8 days after eclosion were immersed in 10% formaldehyde solution for 12 h. Fixed samples were then dehydrated in an ethanol series of 50%, 75%, 90%, and 100%, with at least 30 min in each step. Following dehydration, samples were cleared with xylene. Cleared samples were embedded in paraffin and sectioned. Sections were dewaxed with xylene and ethanol, hydrated, and stained with hematoxylin and eosin. After staining, samples were dehydrated with anhydrous alcohol for 30 s and 1 to 2 drops of neutral glue were added to seal the samples. Samples were observed and photographed under a microscope. Each treatment included three biological replicates of five locust individuals each.

### *L. migratoria manilensis* reproductive and developmental indices

In controls and groups infected with *P. locustae*, the length and weight of egg pods and the length and width of each egg were measured. Each treatment included three biological replicates of eight individuals each. In an additional set of experiments, amount of oviposition was measured in the two treatments and control. Each treatment included three biological replicates of eight individuals each. After hatching, offspring were fed individually in transparent plastic feeding tubes and maintained in an artificial climate chamber. Each treatment included more than 30 individuals. Locust nymph development was recorded twice a day at 8:00 AM and 8:00 PM.

### Statistical analyses

Statistical analyses were performed with the GraphPad Prism 8.3.0 software. Except for the data from sections of ovarian tissue, statistical significance analysis of the remaining data presented in this study were analyzed using one-way or two-way ANOVA followed by Tukey’s multiple comparisons test. The confidence interval was set to 95%, and *P < 0.05* was considered statistically significant.

## Results

### Changes in food Intake of locusts infected with *P. locustae*

**Figure 1:**
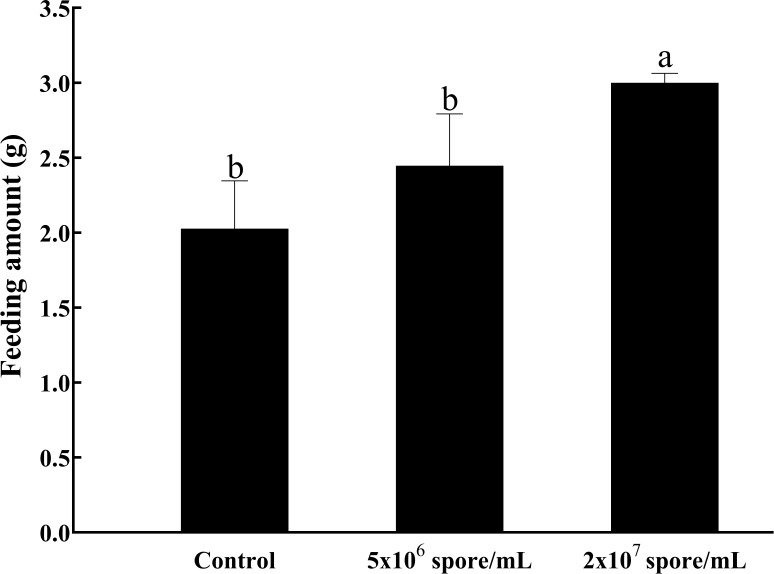
Weight (g) of food consumed by *L. manilensis* after infection with *P. locustae*. Average daily food intake of adult females on days 1–4 after eclosion. Each value is presented as mean ± SD, and different letters indicate significant differences among different treatments (one-way ANOVA followed by Tukey’s multiple comparisons test, *P* < 0.05).

To understand changes in *L. migratoria manilensis* metabolism after microbial infection, effects of infection with different concentrations of *P. locustae* on locust feeding behavior were determined. Compared with uninfected control females, infected locusts consumed more food (*F*_2,6_ = 9.53; *P* = 0.0137). When infected with a concentration of 2 × 10^7^ spores/mL, food intake increased significantly and was more than 1.48-fold higher than that in the control (**Figure 1**).

### Changes in enzyme activity in locusts Infected with *P. locustae*

**Figure 2:**
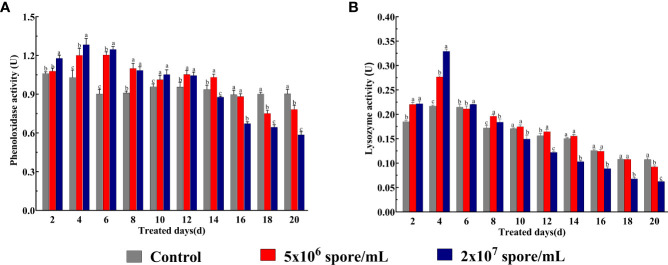
Phenol oxidase (PO) and lysozyme activities in *L*. *migratoria manilensis* infected with *P. locustae.* **(A)** PO activity of adult females were infected by *P. locustae* on days 2–20. **(B)** Lysozyme activity of adult females infected by *P. locustae* on days 2–20. Each value is presented as mean ± SD, and different letters indicate significant differences among different treatments at the same time point (two-way ANOVA followed by Tukey’s multiple comparisons test, *P*  <  0.05).

To understand changes in immune function in *L. migratoria manilensis* infected by the fungus, PO and lysozyme activities were examined. According to two-way ANOVA, PO activity was significantly affected by *P. locustae* concentration (*F*_9,150_ = 425.8; *P* < 0.001), duration of infection (*F*_2,150_ = 67.77; *P* < 0.001), and their interaction (*F*_18,150_ = 89.78; *P* < 0.001). PO activity in 5 × 10^6^ and 2 × 10^7^ spores/mL treatments was higher than that in the control from day 2 to 12 but was significantly lower from day 16 to 20. PO activity was 1.33-fold and 1.38-fold higher in 5 × 10^6^ and 2 × 10^7^ spores/mL treatments, respectively,than that in the control at 6 days (**Figure 2A**). According to two-way ANOVA, lysozyme activity was significantly affected by *P. locustae* concentration(*F*_9,150_ = 4,294; *P* < 0.001), duration of infection (*F*_2,150_ = 323.3; *P* < 0.001), and their interaction (*F*_18,150_ = 272; *P* < 0.001). Compared with the control, microsporidian infection increased lysozyme activity from day 2 to 8 but decreased it from day 10 to 20. Lysozyme activity was strongly induced at 4 days and was 1.28-fold (5 × 10^6^ spores/mL) and 1.52-fold (2 × 10^7^ spores/mL) higher than that in the control. Lysozyme activity at the high concentration (2 × 10^7^ spores/mL) began to decrease significantly by day 10. However, enzyme activity at the low concentration (5 × 10^6^ spores/mL) was not significantly different from that in the control from day 10 to 18 (**Figure 2B**). Therefore, a microsporidian solution with a concentration of 2 × 10^7^ spores/mL led to the greatest stimulation in enzyme activity (**Figure 2**).

### Changes in expression of Vg and VgR genes in locusts Infected with *P. locustae*

**Figure 3:**
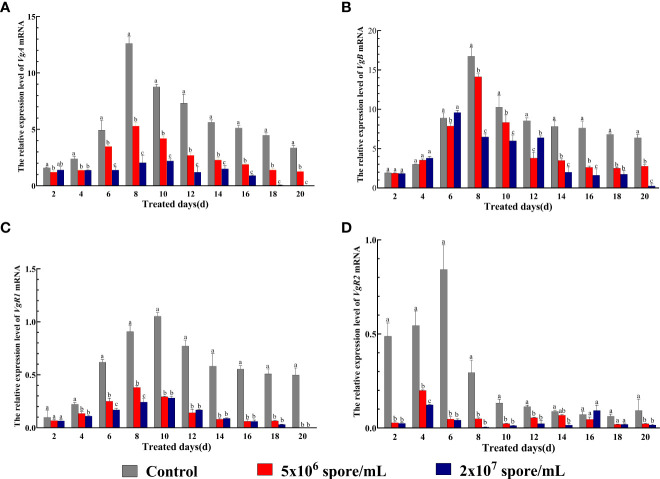
Expression of on *Vg* and *VgR* genes in expression after *L. migratoria manilensis* was infected by *P. locustae*. **(A)** Relative expression of *VgA* mRNA in adult females infected by *P. locustae* on days 2–20. **(B)** Relative expression of *VgB* mRNA in adult females infected by *P. locustae* on days 2–20. **(C)** Relative expression of *VgR1* mRNA in adult females infected by *P. locustae* on days 2–20. **(D)** Relative expression of *VgR2* mRNA in adult females infected by *P. locustae* on days 2–20. Each value is presented as mean ± SD, and different letters indicate significant differences among different treatments at the same time point (two-way ANOVA followed by Tukey’s multiple comparisons test, *P*  <  0.05).

To examine effects of *P. locustae* infection on locust reproductive capacity, mRNA expression of *VgA* and *VgB* and their receptors *VgR1* and *VgR2* was examined. Two-way ANOVA showed that expression of *VgA*, *VgB*, *VgR1*, and *VgR2* was significantly affected by *P. locustae* concentration (*VgA*: *F*_2,90_ = 1871, *P* < 0.001; *VgB*: *F*_2, 90_ = 480.9, *P* < 0.001; *VgR1*: *F*_2, 90_ = 1987, *P* < 0.001; *VgR2*: *F*_2, 90_ = 531.8, *P* < 0.001), duration of infection(*VgA*: *F*_9,90_ = 310.3, *P* < 0.001; *VgB*: *F*_9, 90_ = 398.6, *P* < 0.001; *VgR1*: *F*_9, 90_ = 210.3, *P* < 0.001; *VgR2*: *F*_9, 90_ = 98.32, *P* < 0.001), and their interaction (*VgA*: *F*_18,150_ = 83.71, *P* < 0.001; *VgB*: *F*_18, 90_ = 51.18, *P* < 0.001; *VgR1*: *F*_18, 90_ = 51.91, *P* < 0.001; *VgR2*: *F*_18, 90_ = 67.63, *P* < 0.001) (**Figure 3**). Expression of *VgA* and *VgR1* began to decrease significantly by day 4, and expression was significantly inhibited at 2 × 10^7^ spores/mL at day 20 and was 20820.9-fold and 38111.9-fold lower, respectively, than that in the control (**Figures 3A, C**). Compared with control females, *VgB* expression was down-regulated on day 8 (**Figure 3B**) with *P. locustae* treatment. However, expression of *VgR2* was significantly suppressed from day 2 (**Figure 3D**). The results indicated that *P. locustae* inhibited the transcription of *Vg* and *VgR* genes in locusts.

### Changes in ovaries in locusts infected with *P. locustae*

**Figure 4:**
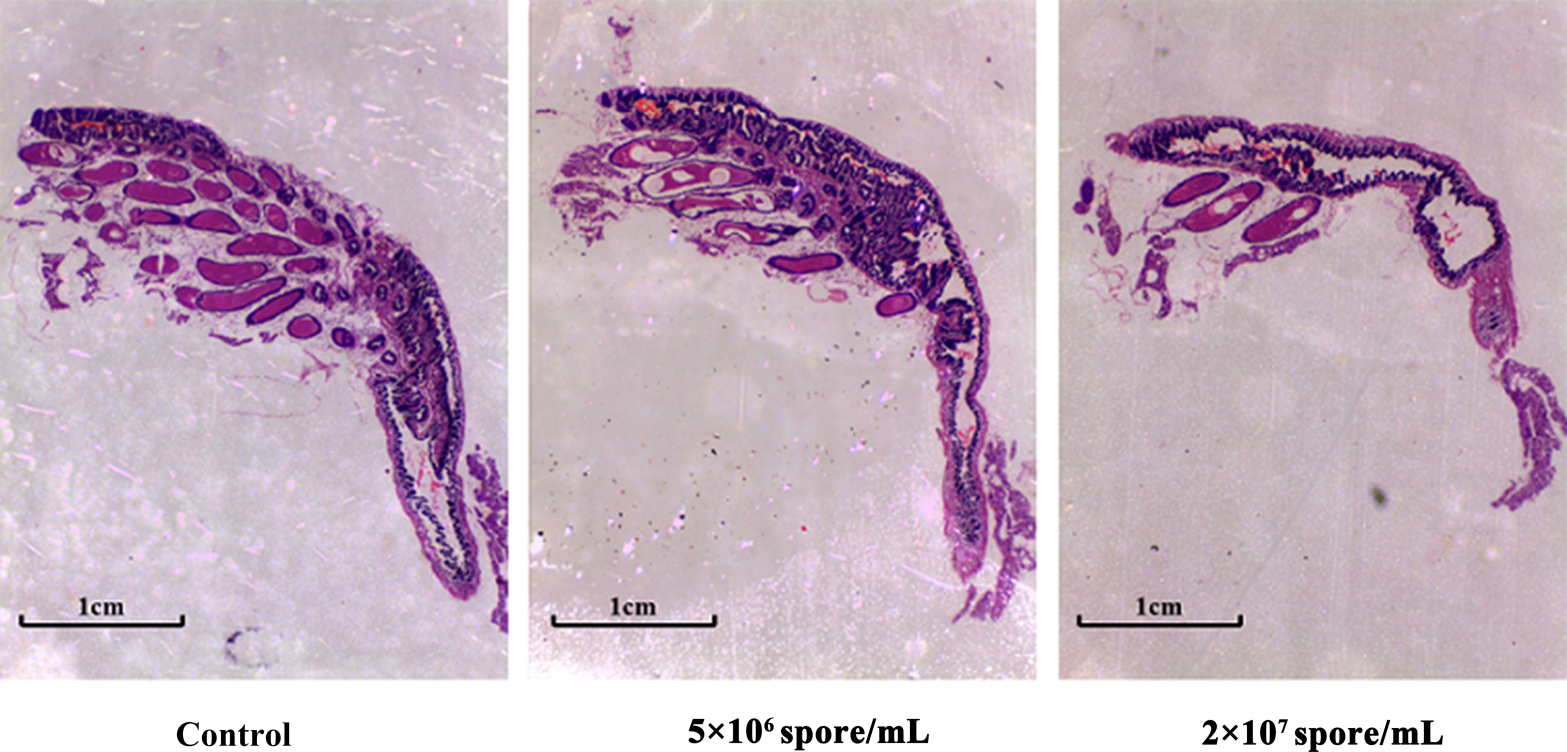
Effects of different spore concentrations of *P. locustae* on ovary development in *L. migratoria manilensis*. Longitudinal sections of locust ovarian tissue on day 8 after eclosion. Ovarian tissues were fixed with formaldehyde solution and then dehydrated in an ethanol series. After clearing with xylene, the samples were embedded in paraffin and stained with hematoxylin and eosin (H&E). Samples were observed and photographed under amicroscope. Scale bar = 50 μm.

Compared with control females, ovary size and number of well-developed eggs decreased slightly after day 8 at 5 × 10^6^ spores/mL, but decreased significantly at 2 × 10^7^ spores/mL (**Figure 4**). Therefore, ovarian development was abnormal with severe atrophy in locusts infected with *P. locustaea*.

### Changes in number and quality of eggs offspring development time in locusts infected with *P. locustae*

**Figure 5:**
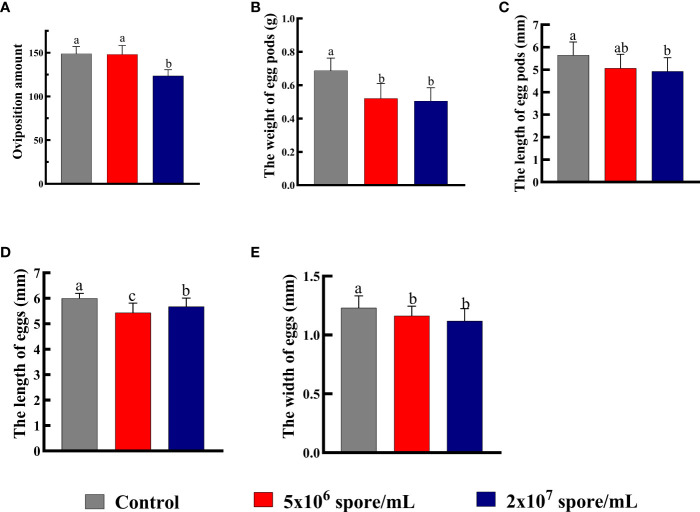
Number and quality of eggs laid by *L. migratoria manilensis* infected with *P. locustae.* **(A)** Oviposition amount or total number of eggs laid by a single adult female. **(B)** Average weight of egg pods laid by a single adult female. **(C)** Average length of egg pods laid by a single adult female. **(D)** Average length of eggs laid by a single adult female. **(E)** Average width of eggs laid by a single adult female. Each value is presented as mean ± SD, and the different letters indicate significant differences among different treatments (one-way ANOVA followed by Tukey’s multiple comparisons test, *P* < 0.05).

**Figure 6:**
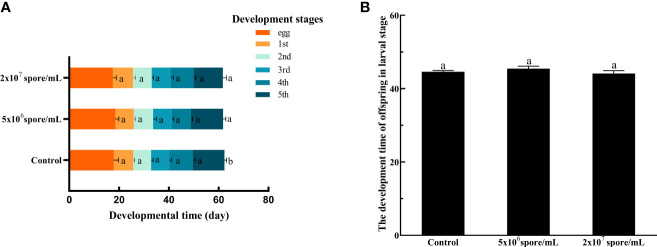
Development time of nymphs after *L. migratoria manilensis* infected with *P. locustae*. **(A)** Development time of each instar: egg, egg stage;1st, 1st instar nymph;2nd, 2nd instar nymph; 3rd, 3rd instar nymph; 4th, 4th instar nymph; 5th, 5th instar nymph. **(B)** Total time for offspring to develop from egg to adult. Each value is presented as mean ± SD, and different letters indicate significant differences among treatments (one-way ANOVA followed by Tukey’s multiple comparisons test, *P* < 0.05).

Total number of eggs laid by a single adult female decreased significantly when infected with *P. locustae* at 2 × 10^7^ spores/mL, compared with the control (*F*_2,30_ = 30.83; *P* < 0.001). The low concentration treatment had no effect on oviposition amount, whereas at the high concentration, total number of eggs laid decreased by 17% (**Figure 5A**). Compared with control females, weight of egg pods (*F*_2,27_ = 15.27, *P* < 0.001; **Figure 5B**) and length of egg pods (*F*_2,27_ = 4.01, *P =* 0.03; **Figure 5C**) decreased significantly in females infected with *P. locustae*. In addition, length (*F*_2,87 =_ 10.48; *P* < 0.001) and width (*F*_2,87_ = 25.78; *P* < 0.001) of eggs decreased significantly after *P. locustae* infection (**Figures 5D, E**). Infection of females with *P. locustae* did not significantly affect offspring development time from egg to adult (*F*_2, 44_ = 1.09, *P* = 0.34; **Figure 6B**). Only in the high concentration treatment, the time required for the 5th instar larvae to develop to adults was significantly shorter than that in the control (*F*_2,44_ = 4.21, *P* = 0.021; **Figure 6A**).

## Discussion

The focus on trade-offs between reproduction and immunity in orthopteran has been on crickets. In dimorphic crickets, the immune response has a stronger effect on male sperm production than on female fertility (33–35), and there is an apparent lack of both physiological trade-offs and terminal reproductive investment in female crickets (36). However, similar studies on locusts have not been reported. In this study, lysozyme and PO activities in female *L. migratoria manilensis* infected with *P. locustae* were examined to assess the cost of immunity against pathogen invasion, and expression of vitellogenin, ovarian development, and development of offspring were examined to assess the reproductive changes in females when resources were invested in immunity.

*P. locustae*, first reported in desert locusts (37), has been used as a biological agent for pest control because it is harmless to humans and livestock (28). Infection with *P. locustae* activates host immunity, and then, insects must invest energy and resources to resist pathogen infection (38). In this study, after infection with *P. locustae*, feeding by locusts increased (**Figure 1**), indicating an increased need for energy to resist pathogens. Glycogen and fat are important energy reserves in insects and are primarily stored in the fat body. Microsporidia invade from the midgut and multiply in the fat body. Transcriptome profiles of *P. locustae* differ significantly in the fat body and midgut during middle and late stages of infection (2). In addition, late in the infection, the load of microsporidia in the fat body is much larger than that in the midgut, indicating the fat body is the eventual site of *P. locustae* eventually multiplication, and nodules form consisting of melanin deposits around heavily infected cells (2). Thus, microsporidia infection damages the fat body (39). After infection with microsporidia, locust glycogen and fat reserves are depleted, and there is rapid uptake of glucose by infected cells (7). However, because pathogens use energy from their hosts, an increase in food consumption may not necessarily increase host immunity (40). Insects lack the acquired immunity and therefore must rely on their innate immunity to resist the pathogens. In insects, the PO cascade, which induces expression of antimicrobial peptides, including lysozyme, is an important part of the humoral immunity (41). PO and prophenol oxidase are found in the sera of most arthropods, including crustaceans and insects, and are essential in defense responses. Insects can also resist bacterial infection through lysozyme shear activity (42).

In this study, PO activity increased significantly from day 2 to 12 (**Figure 2A**), whereas lysozyme activity increased from day 2 to 8 (**Figure 2B**). Those results demonstrated that infection with *P. locustae* activated expression of phenol oxidase and lysozyme, which could increase *P. locustae* mortality. However, PO activity decreased significantly from day 16 to 20, and lysozyme activity decreased significantly from day 10 to20, which indicated that expression of phenoloxidase and lysozyme was inhibited in the late stage of *P. locustae* infection. Reductions in melanization and lytic action against pathogens in locusts might be related to *P. locustae* ability to evade host immunity and contribute to rapid pathogen reproduction in the fat body. In many species, expression of host immunoreactive substances and level of immunity decrease in late phases of pathogen infection. For example, PO activity in honeybees infected with *Spiroplasma melliferum* increases initially and then decreases, and the decrease is associated with evasion of host immune defense by *S. melliferum* to improve its proliferation environment (43), and PO activity decreases sharply in the late stage of *Charybdis japonica* infection with *Vibrio alginolyticus* (44). In *Spodoptera exigua*, lysozyme and other antibacterial active substances are inhibited in the later stage of infection (45). Inhibitory effects of microsporidia on host antibacterial substances have also been observed in honeybees (46, 47). Therefore, microsporidia inhibition of PO the lysozyme activities in locusts could reduce melanization and enable parasites to escape immune responses and survive and proliferate.

Immunity and reproduction both consume large amounts of energy. In this study, female *L. migratoria manilensis* were infected with *P. locustae* early after eclosion, which might affect physiological indices related to reproduction. Activation of the immune system affects fecundity, which is a trade-off in resource allocation observed in many insects. For example, in fruit flies, activation of immunity reduces reproductive output (48). In *Anopheles gambiae*, immunization can induce melanization or humoral antimicrobial activity, and as a result, apoptosis begins in ovarian follicle cell epithelium, leading to a decrease in number of eggs produced (49). In crickets, immune responses can affect female survival rate and egg size (20). In this study, *Vg* expression levels in female *L. migratoria manilensis* decreased significantly with pathogen infection (**Figures 3A, B**), which would inhibit vitellin synthesis. In addition, expression of *VgR* genes decreased significantly (**Figures 3C, D**), which can result in abnormal deposition of vitellin and delayed ovarian development (50, 51). The results suggest that locusts increase energy investment in maintaining fluid circulation and survival after infection and therefore reduce investment in reproduction. In this study, ovaries of microsporidian-treated locusts showed significant atrophy (**Figure 4**), and as the concentration of *P. locustae* increased, the number of eggs laid decreased (**Figure 5A**).

Eggs size reflects reproductive input to offspring and is an important factor in matrilineal inheritance. *In vitro*-fertilized insects sperm combines with relatively large egg cells, and egg size determines an adaptability of the filial generation (52). Although the evolutionary importance of egg size has been debated, growth and survival rates and sometimes fecundity increase in large offspring (53). Resilience to environmental pressures, including larval competition (54), starvation (55, 56), drought (57) and nutritional stress (58, 59), also increases in large offspring. By contrasts, relatively small eggs tend to hatch faster but at a lower survival rate (54, 60). Egg production is also a measure of female fecundity because represents the number of offspring (61, 62). In this study, number of eggs laid (**Figure 5A**) and weight of egg pod (**Figure 5B**) decreased significantly after microsporidian infection. Egg length and width decreased significantly when the concentration reached 2 × 10^7^ spores/mL (**Figures 5D, E**). Therefore, microsporidia infection decreased female fertility, and the decrease was related to microsporidia concentration. Development time of offspring in the 2 × 10^7^ spores/mL treatment group was 1.02 days shorter than that of control offspring (**Figure 6**). This results suggested that high concentrations of *P. locustae* reduced time to hatch.

In this study, decreased expression of *Vg* and *VgR* in *L. migratoria manilensis* infected with *P. locustae* inhibited of vitelline deposition and ovarian development. Amount of oviposition (eggs per pod) decreased significantly, and nutritional status of eggs also decreased. Overall, the results of this study suggest the immune response to *P. locustae* reduced energy resources required for locust reproduction. In addition, new ideas were generated in this study for further studies on molecular mechanisms of the trade-offs between immunity and reproduction in locusts. Further studies are warranted on allocation of energy resources between immune responses and reproductive output and on identification of potential regulatory mechanisms, including energy-metabolizing and nutrient signaling, that could be targets for control of *L. migratoria*.

## Data availability statement

The original contributions presented in the study are included in the article/supplementary material. Further inquiries can be directed to the corresponding authors.

## Author contributions

S-GW and BT designed the work. Y-WH, G-QX, Y-JD and Q-YX carried out the experiments. Y-WH, S-HW, and YT performed the analysis. LZ provides *Paranosema locustae* for this research. Y-WH wrote the initial manuscript. S-HW, BT, LZ, S-GW and YT involved in interpreting data and revising manuscript. All authors read and approved the final manuscript.

## Funding

This work was supported by National Key Research and Development Program of China (Grant No. 2017YFD0201000) and National Natural Science Foundation of China (Grant Nos. 31270459).

## Acknowledgments

Thanks to China Agricultural University for providing Paranosema locustae for this research.

## Conflict of interest

The authors declare that the research was conducted in the absence of any commercial or financial relationships that could be construed as a potential conflict of interest.

## Publisher’s note

All claims expressed in this article are solely those of the authors and do not necessarily represent those of their affiliated organizations, or those of the publisher, the editors and the reviewers. Any product that may be evaluated in this article, or claim that may be made by its manufacturer, is not guaranteed or endorsed by the publisher.

## Abbreviations

VgA, vitellogenin A; VgB, vitellogenin B; VgR, vitellogenin receptor; PO, phenol oxidase; RT-qPCR, reverse-transcription quantitative polymerase chain reaction.
